# Cognitive activity of infants after severe brain damage (early habilitation/ rehabilitation)

**DOI:** 10.1192/j.eurpsy.2024.407

**Published:** 2024-08-27

**Authors:** A. Zakrepina, D. Martishevskaya, Y. Sidneva

**Affiliations:** ^1^ Clinical and Research Institute of Emergency Pediatric Surgery and Trauma (CRIEPST); ^2^Federal state budgetary scientific institution “Institute of correctional pedagogy”/ ICP/; ^3^N.Burdenko National Medical Research Center of Neurosurgery, Moscow, Russian Federation

## Abstract

**Introduction:**

One of the forms of early comprehensive care for children after severe brain injuries is inpatient habilitation/ rehabilitation. Children receive help from a team of medical, psychological and pedagogical specialists. The process of special education consists mainly in the development of cognitive interest, because it is the basis of socialization.

**Objectives:**

To study cognitive activity in children who have suffered severe brain damage.

**Methods:**

observation, pedagogical examination, psychiatric supervision.

**Materials:**

36 children aged 1.2-1.8 years during hospital treatment.

**Results:**

According to the results of the pedagogical survey, three groups of children were identified.

Group 1 (11%): fixed gaze; emotional response to sound (smile); short-term eye tracking of an object; ability to touch an object and hold it for a short time; walking skill is formed.

Group 2 (33%): short-term gaze fixation; reaction to sound by involuntary hand movements; lack of eye tracking of an adult’s face; lack of ability to touch or hold an object; walking skill is formed.

Group 3 (56%): lack of fixed gaze; reaction to sound by shouting and increased motor activity; lack of ability to touch or hold an object; lack of walking skills.

**Conclusions:**

Indicative responses to an adult’s voice and face, eye tracking of an object, sensorimotor activity, and so on. these are indicators that show whether a child has cognitive activity. The rehabilitation team can rely on these indicators when choosing treatment and the content of the special educational process.

***Key words:** early intervention, toddlers, organic damage of central nervous system, rehabilitation/ habilitation*

**Disclosure of Interest:**

None Declared

